# Efficacy of acupuncture for anxiety and depression in functional dyspepsia: A systematic review and meta-analysis

**DOI:** 10.1371/journal.pone.0298438

**Published:** 2024-03-07

**Authors:** Zhiwei Xu, Xuecheng Zhang, Hongshuo Shi, Minghao Liang, Fenglan Ning, Qi Wang, Hongling Jia

**Affiliations:** 1 School of Acupuncture and Tuina, Shandong University of Traditional Chinese Medicine, Jinan, Shandong, China; 2 Department of Proctology, China-Japan Friendship Hospital, Beijing, China; 3 School of Traditional Chinese Medicine, Shandong University of Traditional Chinese Medicine, Jinan, Shandong, China; 4 Department of Acupuncture and Rehabilitation, Longkou Traditional Chinese Medicine Hospital, Yantai, Shandong, China; 5 Acupuncture Department, Second Affiliated Hospital of Shandong University of Traditional Chinese Medicine, Jinan, Shandong, China; Hong Kong Metropolitan University, HONG KONG

## Abstract

**Objective:**

To assess the effectiveness of acupuncture for treating depression and anxiety in patients diagnosed with functional dyspepsia (FD).

**Methods:**

PubMed, Embase, Cochrane Library, Web of Science, CNKI, Wanfang Data, Sinomed, and VIP Database were searched until April 30, 2023 for Randomized Controlled Trials (RCTs) comparing acupuncture to placebo or drugs for symptom alleviation. Two independent reviewers conducted the study search, data extraction, and bias risk assessment using the Cochrane Risk of Bias tool. Mean difference (MD), risk ratio (RR), and corresponding 95% confidence intervals (CI) were computed. Subgroup and sensitivity analyses were also performed. The Grading of Recommendations Assessment, Development, and Evaluation (GRADE) system was employed to evaluate the evidence level.

**Results:**

A total of 16 RCTs involving 1315 participants were included. Acupuncture demonstrated marked superiority over placebo (MD = -7.07, 95%CI: -11.03 to -3.10, very low quality evidence) in mitigating Self-Rating Anxiety Scale (SAS) scores and was found to be more effective in reducing Self-Rating Depression Scale (SDS) scores than either placebo (MD = -4.63, 95%CI: -6.28 to -2.98, low quality evidence) or first-line drugs (MD = -2.71, 95%CI: -5.19 to -0.23, very low quality evidence). In terms of attenuating Hamilton Anxiety Rating Scale (HAMA) and Hamilton Depression Rating Scale (HAMD) scores, acupuncture consistently outperformed both placebo (HAMA: MD = -2.58, 95%CI: -4.33 to -0.83, very low quality evidence; HAMD: MD = -1.89, 95%CI: -3.11 to -0.67, low quality evidence) and first-line drugs (HAMA: MD = -5.76, 95%CI: -10.18 to -1.35, very low quality evidence; HAMD: MD = -5.59, 95%CI: -7.59 to -3.59, very low quality evidence). However, no significant difference was observed between acupuncture and placebo in terms of improvement in Hospital Anxiety and Depression Scale (HADS) scores.

**Conclusions:**

Based on current clinical evidence, acupuncture might have a positive effect on depression and anxiety in patients with FD. Further large-sample, multi-center, high-quality RCTs validation are required, as the conclusion is limited by the quantity and quality of the included studies.

## Introduction

Functional dyspepsia (FD) manifests as a chronic functional gastrointestinal disorder, distinguished by recurrent episodes of epigastric pain, epigastric burning, early satiety, and postprandial fullness, with no discernable structural alterations upon gastroscopic examination [1, 2]. The underlying pathophysiological mechanisms remain elusive, potentially linked with aberrations in gut-brain communication, thus engendering dyskinesia, visceral hypersensitivity, diminished mucosal function and immunity, and modifications in central nervous system processing [1, 3]. Individuals diagnosed with FD demonstrate a greater susceptibility to anxiety, depression, and other mood disorders when compared to the general population. The prevalence of adverse emotional states amongst Chinese FD patients stands at 15.1%, escalating to an alarming 54.2% amongst hospitalised patients [4]. A longitudinal study over a decade in Sweden revealed that individuals grappling with anxiety disorders exhibited a 7.6-fold increased risk of developing FD after a 10-year period [5]. Notably, evidence have shown there was a significant correlation between anxiety and FD symptoms, with improvements in anxiety linked to a decline in FD symptom severity [6–8]. Therefore, it is imperative to accord increased attention to anxiety and depression within the FD context.

In light of these challenges, first-line drugs are limited in their effectiveness in relieving anxiety and depression. While second-line medications like central neuromodulators can ameliorate these symptoms [3], they come with significant side effects like drowsiness and dependency that should not be overlooked [9, 10]. These negative effects could both impair the patient’s quality of life and lessen adherence to treatment.

Given this backdrop, acupuncture stands out as an essential non-pharmacological treatment option. Implemented in more than 183 countries globally [11], it has shown clear benefits in alleviating mental health issues like anxiety and depression, with almost no side effects [12, 13]. However, despite independent studies investigating the role of acupuncture in FD alongside mental health issues such as anxiety and depression, these studies have not arrived at uniform conclusions [14]. Especially in scenarios where FD and mental health problems are present simultaneously, a thorough assessment of acupuncture’s overall efficacy has yet to be conducted.

Consequently, the goal of this meta-analysis is to bridge the current gap in knowledge. Utilizing existing Randomized Controlled Trials (RCTs) studies, we conducted a meta-analysis to evaluate the clinical effectiveness of acupuncture for managing FD with concomitant anxiety and depression, aiming to provide more robust evidence and unbiased, comprehensive assessments for clinical guidance.

## Methods

### Protocol and registration

The reporting guideline used in this review was the Preferred Reporting Items for Systematic Reviews and Meta-Analyses (PRISMA 2020) [15, 16]. The registration number of the protocol is CRD42022350496 (https://www.crd.york.ac.uk/prospero/).

### Eligibility criteria

RCTs published in English or Chinese were included. Participants of any demographic background, whether by age, sex, or disease duration, with a confirmed diagnosis of FD as per the Rome II, Rome III or Rome IV criteria were eligible for inclusion [17–19]. Participants in the experimental group received solitary acupuncture therapy, with no restriction on the acupoints selected. Acupuncture therapy, premised on the principles of Traditional Chinese Medicine (TCM), involves the stimulation of specific acupuncture points and encompasses methodologies such as traditional acupuncture, electroacupuncture, transcutaneous electrical acupoint stimulation (TEAS), and others. Conversely, the control group was subjected to either placebo acupuncture or conventional drug treatment. The placebo acupuncture was implemented by needling a point located marginally millimeters or centimeters away from the actual acupuncture point or needling a point without penetrating the skin [20]. This was performed to mitigate potential placebo effect and substantiate the validity of the study outcomes [20]. Outcome measures necessitated at least one assessment of anxiety, depression or overall symptoms. Primary outcomes incorporated the Self-Rating Anxiety Scale (SAS) [21], the Self-Rating Depression Scale (SDS) [22], the Hamilton Anxiety Rating Scale (HAMA) [23], the Hamilton Depression Rating Scale (HAMD) [24], and the Hospital Anxiety and Depression Scale (HADS) [25]. A secondary outcome pertained to the global alleviation of symptoms [26, 27].

We excluded reviews, animal trials, theses, conference papers, and any studies without the full text available.

### Information sources and search strategy

We conducted a comprehensive search of multiple databases to identify RCTs investigating the impact of acupuncture on depression and anxiety in patients with FD. The databases included PubMed, Embase, the Cochrane Library, Web of Science, China National Knowledge Infrastructure (CNKI), Wanfang Data, Sinomed, and the VIP Database. The search was carried out from the inception of each database until April 30, 2023. Two independent reviewers (ZX and ML) conducted the search process. The search strategy integrated both Medical Subject Headings (MeSH) terms and free-text words. The terms deployed in the search included "functional dyspepsia", "non-ulcer dyspepsia", "acupuncture", "electroacupuncture", "needle", "randomized controlled trial", and so forth. The PubMed search strategy can be found in S1 Table, as presented in the S1 File.

### Selection process and data extraction

Two reviewers (ZX and ML) independently engaged in the screening of studies and extraction of data. Discrepancies, if any, were addressed through consultation with a third reviewer (HJ). For each study, the data extracted encompassed the following parameters: random sequence generation, allocation concealment, blinding, outcome completeness, first author, publication year, country, language, sample size, age, sex, diagnostic criteria, disease course, interventions, outcomes, treatment duration, and follow-up, among others. In the event of missing crucial information, at least two emails were sent to the corresponding author to respectfully seek the complete data.

### Assessment of risk of bias

The Cochrane Risk of Bias Tool [28] was utilized to evaluate the quality of the studies incorporated. Seven potential sources of bias were scrutinized: random sequence generation, allocation concealment, blinding of participants and personnel, blinding of outcome assessment, incomplete outcome data, selective reporting, and other bias. Each source was determined to be at low risk, unclear risk, or high risk, based on the information disclosed in the respective studies. Two independent reviewers (ZX and ML) undertook this assessment, and the outcomes were subsequently rendered into figures using the RevMan 5.4 software.

### Data analysis

The execution of the meta-analysis was facilitated by the RevMan 5.4 software. For dichotomous outcomes, the relative risk (RR) along with its corresponding 95% confidence interval (CI) were estimated, whereas for continuous outcomes, the mean difference (MD) and its accompanying 95% CI were calculated. The degree of heterogeneity amongst the included RCTs was ascertained using the Q-test’s *P*-value and the *I*^*2*^ statistic. In scenarios where *I*^*2*^ ≤ 50% and *P* ≥ 0.1, indicative of a lack of statistical heterogeneity between studies, a fixed-effects model was employed for data pooling. On the contrary, when *I*^*2*^ > 50% and *P* < 0.1, signifying statistical heterogeneity between studies, a random-effects model was adopted. In the event of heterogeneity, sensitivity analysis and subgroup analysis were initiated to probe potential sources of heterogeneity. To assess the stability of the results, a sensitivity analysis was also undertaken. If sufficient included studies were available, a funnel plot was used to assess publication bias.

### Level of evidence

The Grading of Recommendations Assessment, Development, and Evaluation (GRADE) system [29] was employed to assess the quality of evidence. RCTs were given an initial rating as high-level evidence, subject to possible downgrade based on several factors such as risk of bias, inconsistency, indirectness, imprecision, and the potential presence of publication bias. The quality of the evidence was subsequently evaluated and categorized as high, moderate, low, or very low.

## Results

### Study selection

**Fig 1:**
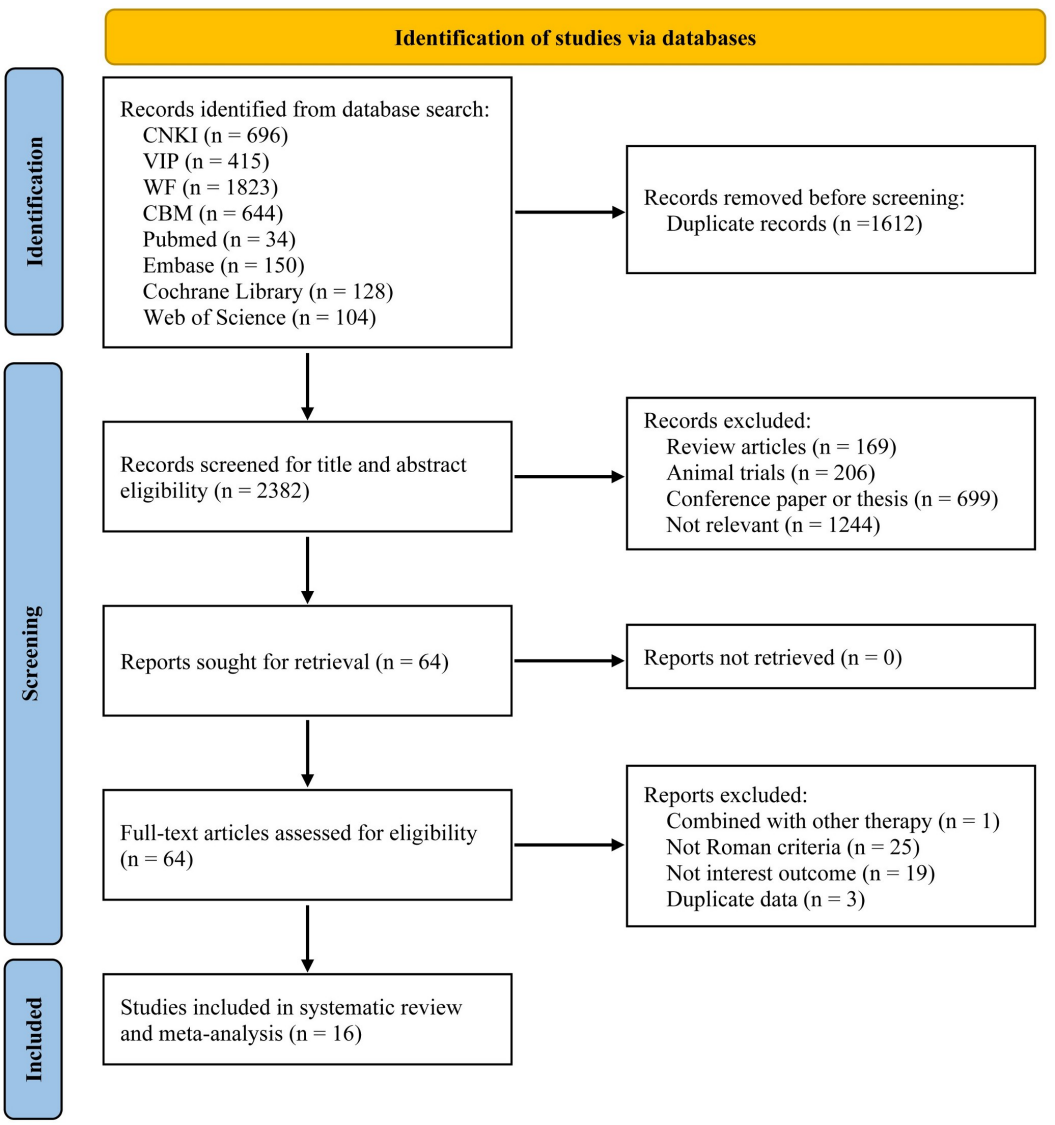
Flow of literature search and selection.

A total of 3994 studies were retrieved. After duplicates were removed and strict inclusion and exclusion criteria were followed for screening, 16 studies were ultimately included in our analysis. For details on the screening process, please refer to Fig 1.

### Study characteristics

Our review incorporated a total of 16 studies, with an aggregate of 1315 participants being examined (Table 1). Among these, four studies [14, 30–32] were conducted in English and twelve [33–44] in Chinese, with all occurrences taking place within China. Five studies [14, 31, 32, 36, 43] adopted the Rome IV diagnostic criteria for assessment, while the remaining eleven used the Rome III criteria. The mean duration of the disease spanned from 0.71 to 12.2 years, and the mean age of the study subjects ranged from 27.43 to 57.53 years. The range of interventions explored included manual acupuncture [14, 30, 33, 37–39, 41–43], electroacupuncture [32, 34, 35, 40, 44], and transcutaneous electrical acupoint stimulation [31, 36]. The control groups comprised of placebo acupuncture [14, 30, 31, 34, 36, 43], first-line drugs [32, 33, 35, 37–40, 44], and a combination of first-line and second-line drugs [41, 42]. Seven studies [14, 30–32, 36, 37, 43, 44] received funding.

**Table 1:** The Characteristics of the included studies.

References	Language	Diagnostic criteria	Mean age (mean disease course) T/C	Sample size (% male) T/C	Intervention		Treatment duration	Follow up	outcome
					T	C			
**Chen XL 2022 [31]**	English	Rome IV	57.53(NR)/ 51.13(NR)	15(47)/ 15(67)	TEAS 3 times a day	Placebo-TEAS 3 times a day	4 weeks	NR	①②
**Du R 2016 [44]**	Chinese	Rome III	44.89(4.67)/ 43.28(4.38)	48(27)/ 47(32)	EA 30min 6 times a week	Domperidone 10mg tid Omeprazole 20mg qd	4 weeks	3 months	③
**Hou YQ 2020 [43]**	Chinese	Rome IV	41.93(5.45)/ 42.5(5.88)	30(33)/ 30(33)	MA 20min 3 times a week	Placebo-MA 20min 3 times a week	4 weeks	NR	⑥
**Jin YL 2015 [30]**	English	Rome III	49.29(12.2)/ 48.25(12.11)	28(39)/ 28(36)	MA 20min 4 times a week	Placebo-EA 20min 4 times a week	4 weeks	3 months	①②
**Liao W 2012 [42]**	Chinese	Rome III	NR(NR)/ NR(NR)	40(NR)/ 40(NR)	MA 30-35min Once a day	Domperidone 10mg tid Paroxetine 20mg qd	1 month	NR	①②
**Liu Y 2023 [32]**	English	Rome IV	47.3(0.71)/ 46.9(0.72)	30(57)/ 30(67)	EA 30min Once a day	Mosapride Citrate 5mg tid	20 days	NR	⑥
**Liu ZX 2019 [41]**	Chinese	Rome III	NR(NR)/ NR(NR)	50(46)/ 50(44)	MA 15-20min Once a day	Domperidone 10mg tid Ranitidine hydrochloride 150mg tid Estazolam 1mg tid	6 weeks	NR	①②⑥
**Qiang LM 2018 [40]**	Chinese	Rome III	43.5(4.6)/ 45.9(4.2)	32(38)/ 32(44)	EA 30min Once a day	Mosapride Citrate 5mg tid	1 month	NR	⑥
**Ren J 2015 [39]**	Chinese	Rome III	38.29(1.3)/ 37.65(1.24)	34(56)/ 34(53)	MA 30min Once a day	Domperidone 10mg tid	2 weeks	2 months	⑥
**Shen LJ 2013 [38]**	Chinese	Rome III	40.83(NR)/ 41.32(NR)	35(43)/ 35(45)	MA 30min Once a day	Domperidone 10mg tid	2 weeks	NR	①②
**Shui DK 2014 [37]**	Chinese	Rome III	42(NR)/ 41(NR)	40(55)/ 40(48)	MA 30min Once a day	Mosapride Citrate 5mg tid	3 weeks	NR	⑥
**Wu D 2021 [36]**	Chinese	Rome IV	50.58(4.63)/ 48.31(4.51)	45(36)/ 45(27)	TEAS 30min 5 times a week	Placebo-TEAS 30min 5 times a week	4 weeks	NR	②③④⑥
**Yang JW 2020 [14]**	English	Rome IV	41.6(4.78)/ 41.2(5.13)	138(28)/ 140(38)	MA 20min 3 times a week	Placebo-MA 20min 3 times a week	4 weeks	3 months	⑤
**Yang JY 2011 [35]**	Chinese	Rome III	37.6(NR)/ 38.4(NR)	30(43)/ 30(50)	EA 30min Once a day	Domperidone 10mg tid	4 weeks	NR	①②
**Yang ZQ 2011 [34]**	Chinese	Rome III	27.5(4.42)/ 27.43(6.41)	31(48)/ 30(40)	EA 30min 5 times a week	Placebo-EA 30min 5 times a week	4 weeks	NR	⑥
**Yuan XX 2015 [33]**	Chinese	Rome III	44.21(2.01)/ 39.21(2.61)	31(42)/ 32(47)	MA 40min Once a day	Domperidone 10mg tid	1 month	NR	③④⑥

NR: not report; T: treatment group; C: control group; MA: manual acupuncture; TEAS: transcutaneous electrical acupoint stimulation; EA: electroacupuncture; tid: three times a day; qd: once a day; ①: Self-Rating Anxiety Scale; ②: Self-Rating Depression Scale; ③: Hamilton Anxiety Rating Scale; ④: Hamilton Depression Rating Scale; ⑤: Hospital Anxiety and Depression Scale; ⑥: global symptoms

### Risk of bias in studies

**Fig 2:**
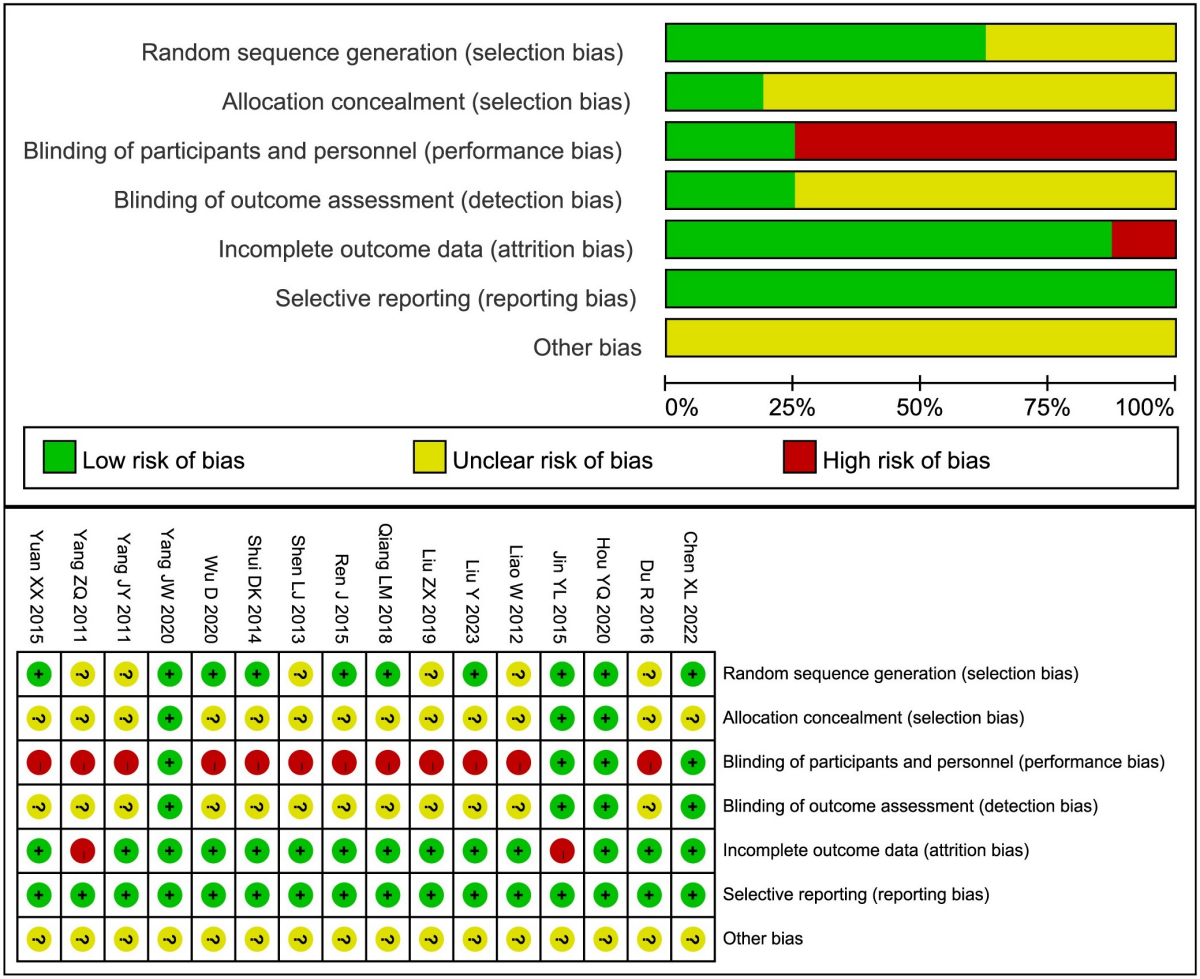
Risk of bias graph and summary.

Ten studies [14, 30–33, 36, 37, 39, 40, 43] employed random number tables or computer software to generate random sequences, which implies a low risk of bias concerning randomization. However, due to insufficient information provided, the risk of bias in the remaining studies remained ambiguous. In terms of selection bias, detailed methodological descriptions were provided in three studies [14, 30, 43], thus classifying them as low risk, whereas the risk remained uncertain in the others. Blinding the experimental process poses a challenge in acupuncture therapy, and a mere four studies [14, 30, 31, 43] were successful in implementing blinding procedures, thereby qualifying as low risk. Outcome analysis blinding was reported in four studies [14, 30, 31, 43], earning them a classification of low risk, while the risk was assessed as unclear in the remaining studies. In the examined pool of four studies [14, 30, 34, 43], attrition was evident. Of these, two studies [30, 34] employed per-protocol (PP) analysis and were consequently categorized as carrying a high risk of bias. Conversely, the remaining two studies [14, 43], which utilized intention-to-treat (ITT) analysis, were deemed to possess a low risk. All studies reported their predetermined outcomes, which suggests a low risk of bias in this aspect. However, with regards to other potential sources of bias, the risk assessment remained unclear across all studies (Fig 2).

### Meta-analysis

#### The SAS scores

**Fig 3:**
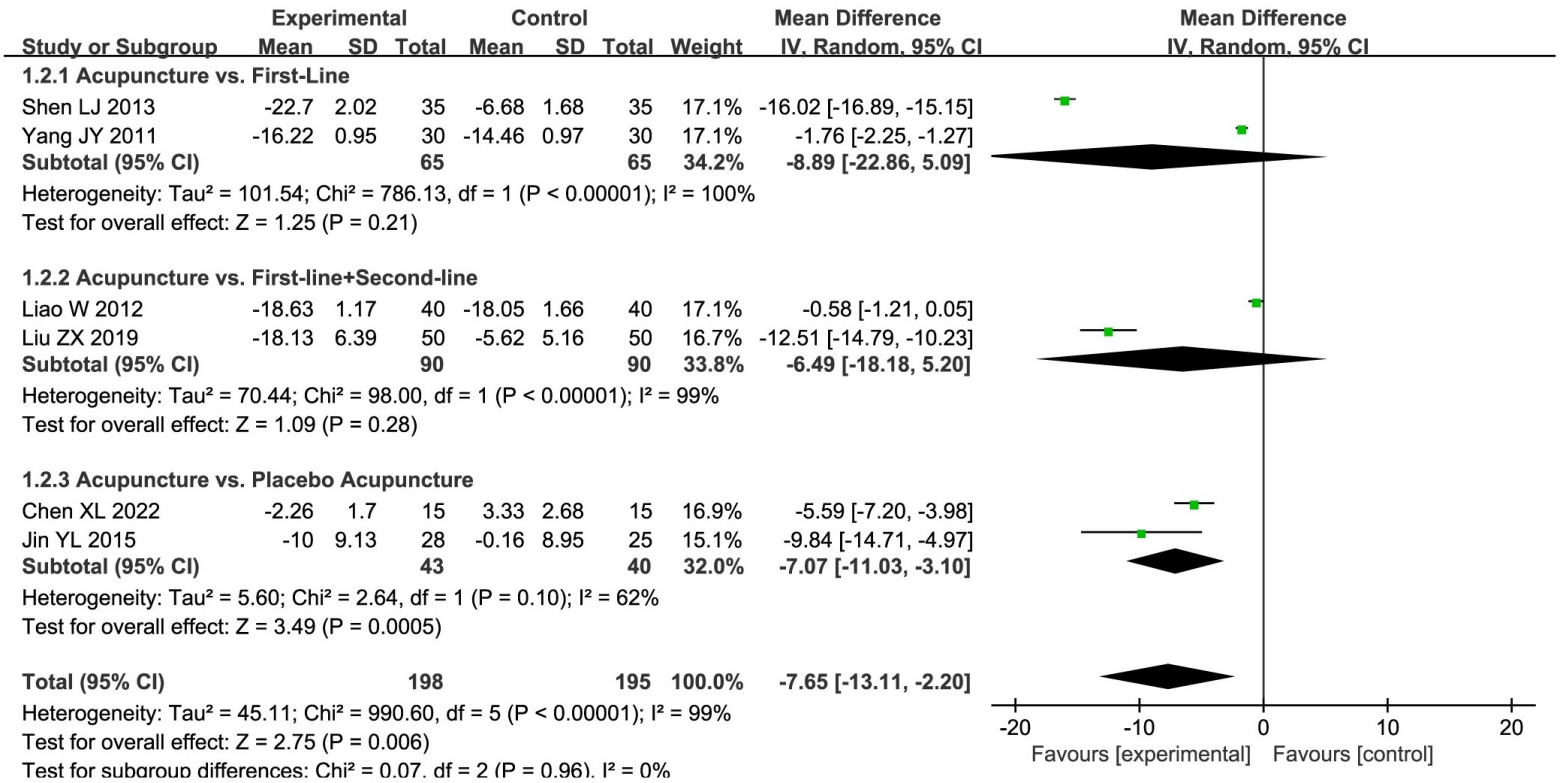
Forest plot of SAS in subgroup analyses.

Six studies [30, 31, 35, 38, 41, 42] documented the SAS scores. A subsequent meta-analysis, employing random-effects model, revealed a more pronounced efficacy in the acupuncture group as compared to the control group (MD = -7.65, 95%CI: -13.11 to -2.20, *P* < 0.00001; *I*^2^ = 99%). A detailed subgroup analysis was subsequently conducted, taking into account the different types of control measures employed, with the corresponding results depicted in Fig 3. The results distinctly suggest that the efficacy of acupuncture in improving SAS scores was significantly greater than that of placebo acupuncture (MD = -7.07, 95%CI: -11.03 to -3.10, *P* = 0.0005; very low quality evidence), underscoring a statistically significant difference. Interestingly, no significant divergence was discerned between the impacts of acupuncture and the administration of first-line drugs alone (MD = -8.89, 95%CI: -22.86 to 5.09, *P* = 0.21; very low quality evidence), or in association with second-line drugs (MD = -6.49, 95%CI: -18.18 to 5.20, *P* = 0.28; very low quality evidence). The aggregated outcomes manifested a certain degree of statistical heterogeneity.

#### The SDS scores

**Fig 4:**
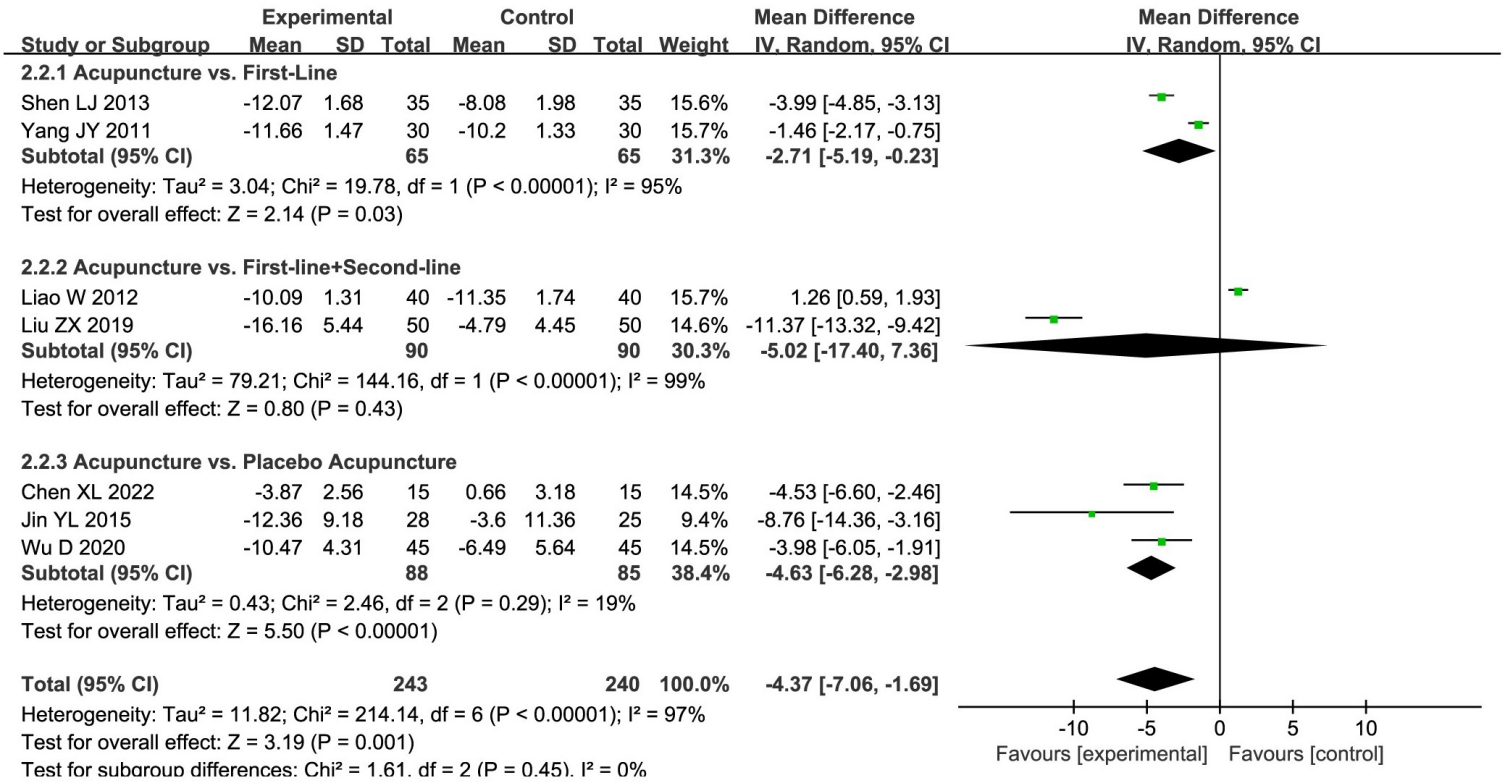
Forest plot of SDS in subgroup analyses.

Seven studies [30, 31, 35, 36, 38, 41, 42] delineated the SDS scores, following which a comprehensive meta-analysis was undertaken to evaluate the efficacy of acupuncture. A meta-analysis employing random-effects model provided evidence of the significant superiority of the acupuncture group relative to the control group (MD = -4.37, 95%CI: -7.06 to -1.69, *P* < 0.00001; *I*^*2*^ = 97%), with significant differences being discernible, despite the presence of certain heterogeneity. To delve deeper into the origins of observed heterogeneity, a meticulous subgroup analysis was subsequently performed (Fig 4). The resultant findings illustrated that acupuncture exhibited a significantly enhanced effectiveness as compared to both placebo acupuncture (MD = -4.63, 95%CI: -6.28 to -2.98, *P* <0.0001; low quality evidence) and first-line drugs (MD = -2.71, 95%CI: -5.19 to -0.23, *P* = 0.03; very low quality evidence). However, no significant differentiation was identified between the effectiveness of acupuncture and the combined usage of first-line and second-line drugs (MD = -5.02, 95%CI: -17.41 to 7.36, *P* = 0.43; very low quality evidence). Despite these observations, the drug group still exhibited a persistent statistical heterogeneity.

#### The HAMA scores

**Fig 5:**
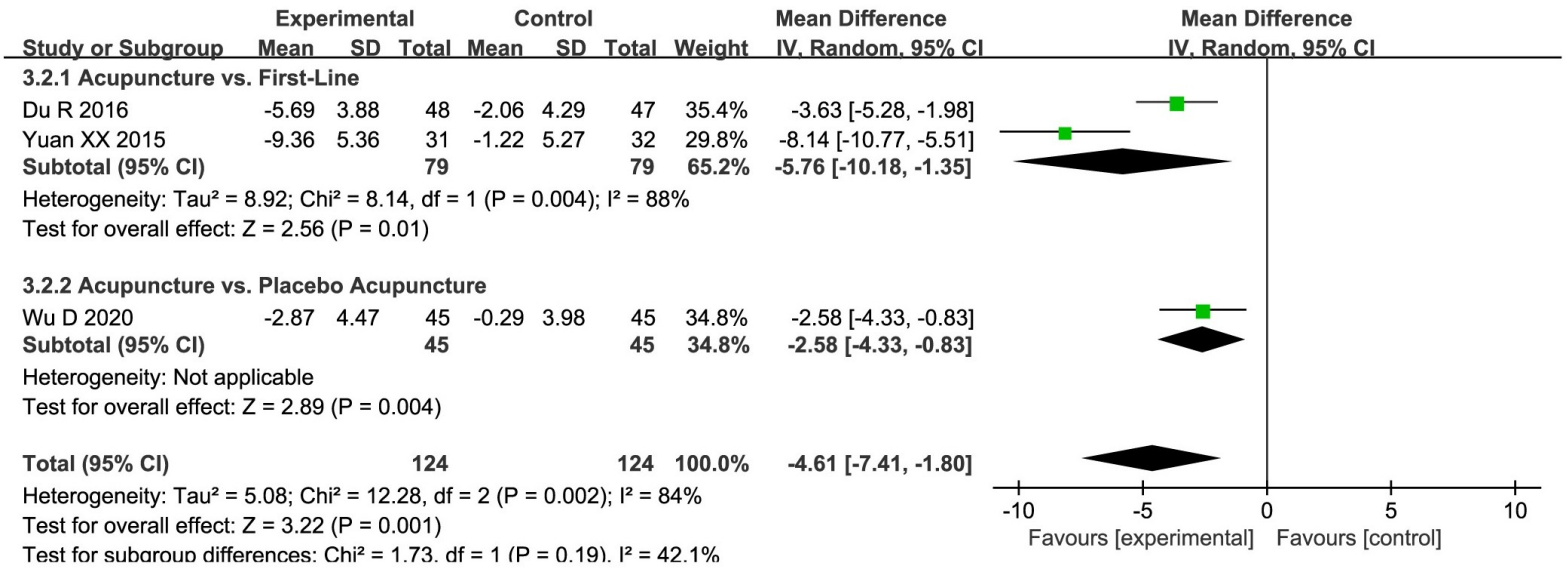
Forest plot of HAMA in subgroup analyses.

Three studies [33, 36, 44] contributed HAMA scores, and a conducted heterogeneity test revealed the presence of significant heterogeneity. Upon executing a random-effects meta-analysis, it was discerned that the acupuncture group (MD = -4.61, 95%CI: -7.41 to -1.80, *P* = 0.002; *I*^*2*^ = 84%) exhibited superiority over the control group in terms of reducing HAMA scores, with the difference being of statistical significance. A subsequent detailed subgroup analysis elucidated that acupuncture was demonstrably more effective in comparison to either placebo acupuncture (MD = -2.58, 95%CI: -4.33 to -0.83, *P* = 0.004; very low quality evidence) or first-line drugs (MD = -5.76, 95%CI: -10.18 to -1.35, *P* = 0.01; very low quality evidence) for the reduction of anxiety, as gauged by HAMA scores (Fig 5).

#### The HAMD scores

**Fig 6:**
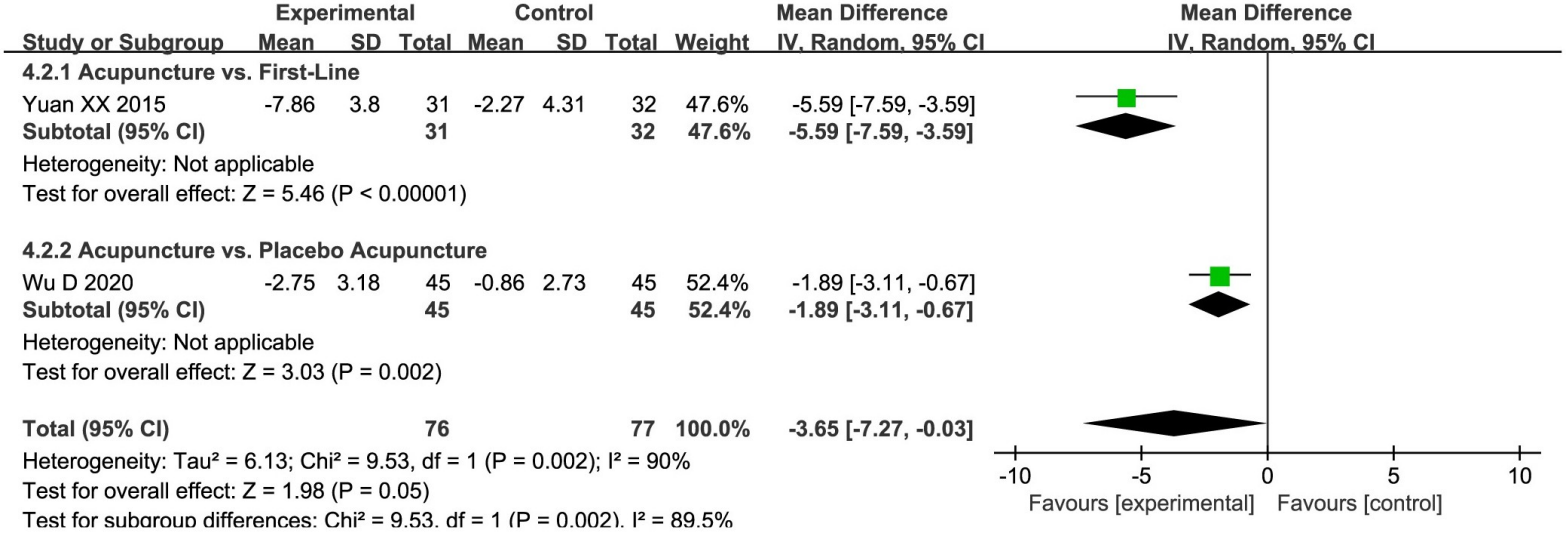
Forest plot of HAMD in subgroup analyses.

An aggregate of two studies [33, 36] proffered the HAMD scores. To juxtapose the efficacy of acupuncture with the control group, a random-effects meta-analysis was undertaken. Despite the significant heterogeneity inherent across the studies, the results illustrated a statistically noteworthy superiority of acupuncture over the control group (MD = -3.65, 95%CI: -7.27 to -0.03, *P* = 0.002; *I*^*2*^ = 90%). Supplemental subgroup analyses further accentuated that acupuncture significantly outperformed both placebo acupuncture (MD = -1.89, 95%CI: -3.11 to -0.67, *P* = 0.002; low quality evidence) and first-line drugs (MD = -5.59, 95%CI: -7.59 to -3.59, *P* < 0.00001; very low quality evidence) in effectiveness (Fig 6).

#### The HADS scores

**Fig 7:**
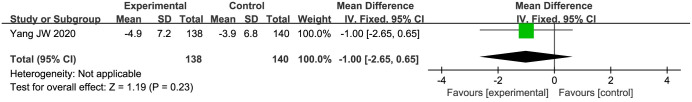
Forest plot of HADS.

The HADS were gleaned solely from one study [14], and the results conveyed no statistically significant disparity between the acupuncture cohort and the placebo acupuncture group (MD = -1.00, 95%CI: -2.65 to -0.65, *P* = 0.23; moderate quality evidence) (Fig 7).

#### Global symptom

**Fig 8:**
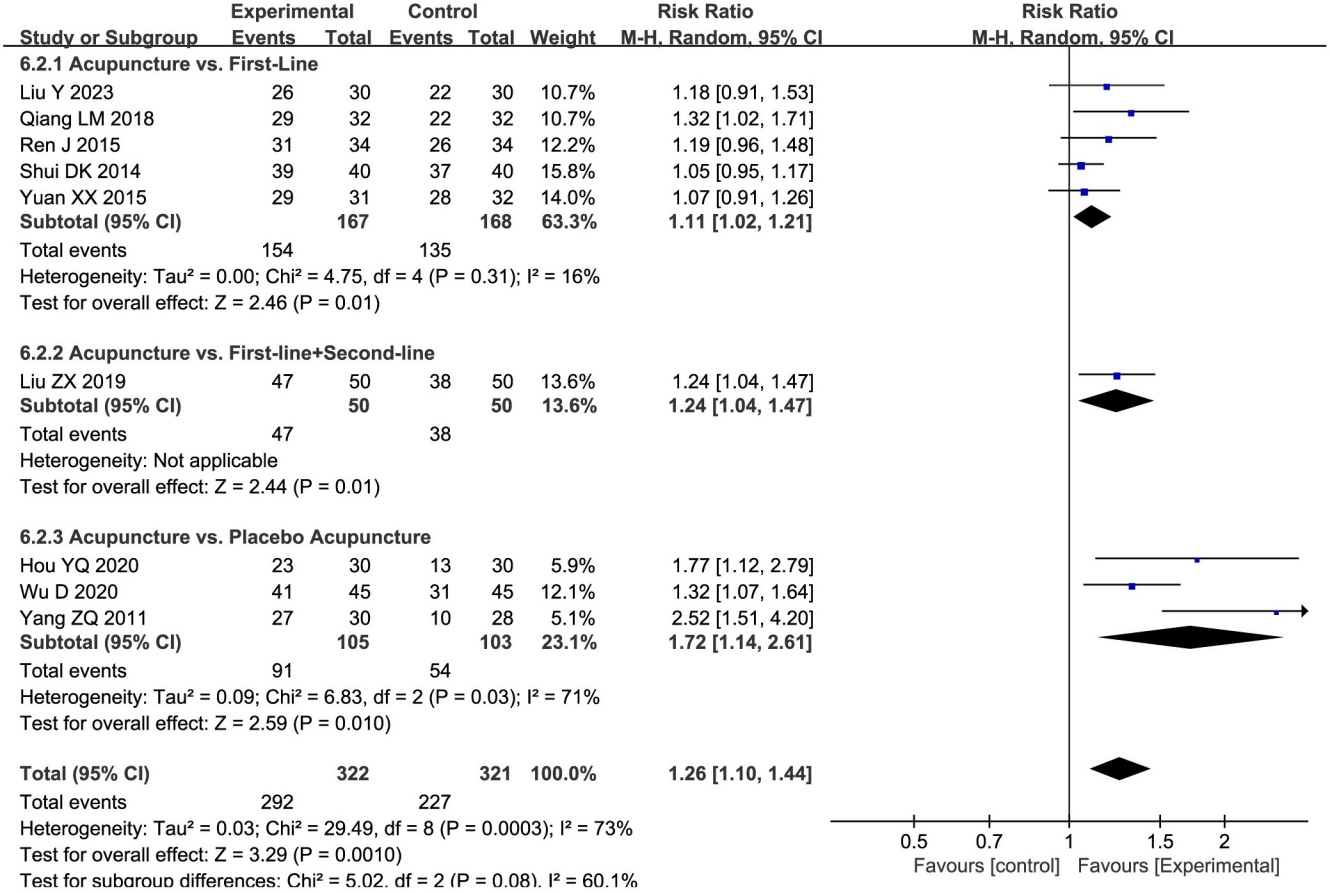
Forest plot of global symptoms in subgroup analyses.

An aggregate of nine studies [32–34, 36, 37, 39–41, 43] enumerated global symptoms. A meta-analysis employing a random-effects model elucidated that the acupuncture group exhibited significant superiority over the control group (RR = 1.26, 95%CI: 1.10 to 1.44, *P* = 0.0003; *I*^*2*^ = 73%). The therapeutic advantage of acupuncture persisted across all subgroups, contingent upon the respective controls. The subgroups were: (1) Placebo-acupuncture (RR = 1.72, 95%CI: 1.14 to 2.61, *P* = 0.03; very low quality evidence), (2) first-line drugs (RR = 1.11, 95%CI: 1.02 to 1.21, *P* = 0.01; low quality evidence), and (3) a combination of first-line drugs with second-line drugs (RR = 1.24, 95%CI: 1.04 to 1.47, *P* = 0.01; very low quality evidence) (Fig 8).

### Sensitivity analysis

Upon the exclusion of a single study [44], it was discerned that the superiority of the acupuncture group over the control group in ameliorating HAMA was no longer manifest, thereby hinting at some potential instability within the outcomes. However, there were no notable changes in all other findings from the sensitivity analysis, thereby confirming the robustness of these results. Additional details can be found in S1-S5 Figs, as presented in the S1 File.

### Publication biases

Following the recommendations from the Cochrane Handbook, it’s cautioned against using the test for funnel plot asymmetry when fewer than 10 studies are included in a meta-analysis [45]. In situations with such limited sample sizes, the statistical power of the test is compromised, making it challenging to differentiate between random variation and genuine asymmetry [46]. Consequently, we refrained from constructing a funnel plot for this analysis.

### Quality of evidence

In the course of our systematic review, we found that a significant proportion of the evaluated evidence was adjudged to be of low or indeed very low quality. This downgrading can be primarily attributed to factors such as Risk of Bias (ROB), manifested in inadequately detailed descriptions of allocation concealment methods and unexplained blinding protocols, inconsistency, denoted by unexplained heterogeneity, and imprecision, marked by overly broad confidence intervals among other issues. A comprehensive account of these concerns is available for further inspection in S6-S11 Figs, as presented in the S1 File.

## Discussion

In this systematic review and meta-analysis of 16 RCTs involving 1315 participants, we assessed the therapeutic efficacy of acupuncture in alleviating anxiety and depression associated with FD, while also noting its positive impact on the symptoms of FD itself. Notably, acupuncture outperformed first-line medications in improving scores on the SDS, HAMA, and HAMD scales, all of which are key indicators for anxiety and depression. Additionally, acupuncture’s effectiveness was comparable to that of combined first-line medications and central neuromodulators in enhancing SAS and SDS scores. More compellingly, in comparisons with placebo acupuncture, genuine acupuncture yielded significant improvements across multiple metrics, namely SAS, SDS, HAMA, and HAMD, thereby affirming its therapeutic legitimacy. Nonetheless, we observed no significant difference in HADS scores when comparing acupuncture with placebo acupuncture. Collectively, our findings emphasize acupuncture’s potential as a viable alternative or complementary treatment for managing anxiety and depression.

This study presents the first systematic meta-analysis that evaluates the efficacy of acupuncture in treating FD accompanied by symptoms of depression and anxiety. Although previous meta-analyses have confirmed the effectiveness of Chinese Herbal Medicine in traditional Chinese medicine for treating FD with psychological conditions [47], no comparable systematic evaluations exist for acupuncture. To bridge this gap, we used various validated scales to measure the severity of depression and anxiety in patients. In compliance with BSG guidelines [48], our study also included recommended central neuromodulators as a control group for comparative analysis. While central neuromodulators are known to have therapeutic benefits, their potential adverse effects often deter both healthcare providers and patients from utilizing these treatments. This limitation underscores the need for acupuncture, a non-pharmacological therapy with a lower risk of side effects. Our results revealed that acupuncture outperformed central neuromodulators on certain scales and even surpassed the combined effectiveness of first-line drugs and central neuromodulators. These findings bolster the case for acupuncture as a viable treatment for FD and its associated emotional disorders, suggesting it could serve as an alternative or supplementary therapeutic option.

However, in our study, we observed inconsistencies across different scales, which we hypothesize may arise from the varying focus of the evaluation tools used. For instance, self-report scales such as SAS [21] and SDS [22] provide a holistic view of both physiological and psychological symptoms, while clinician-assessed scales like HAMA [23] and HAMD [24] concentrate primarily on observable and measurable clinical indicators. This discrepancy lends a credible rationale for the inconsistencies found in our results and suggests that acupuncture may have unique benefits in enhancing patients’ self-perception and specific physiological symptoms.

The pathophysiology of FD is intricately connected to the gut-brain-microbiota axis [49]. This axis plays a crucial role in the interaction between the gastrointestinal tract and the central nervous system. Acupuncture, believed to positively influence this axis, may provide a rationale for its effectiveness in treating anxiety and depression associated with FD. It is theorized that acupuncture helps balance the gut microbiota, thereby improving the intestinal barrier and immune system. This balance could be a key factor in acupuncture’s impact on the gut-brain axis [50]. In contrast to antibiotics, which can have negative effects on gut and mental health, acupuncture is suggested to increase microbial alpha diversity [51, 52]. This diversity may alleviate symptoms of anxiety and depression [51, 52]. Furthermore, acupuncture reduces inflammatory markers like LPS, TNF-α, and IL-1β in serum and brain, which are linked to anxiety and depression, suggesting dual benefits for gut and mental health [53]. Additionally, the modulation of Treg and Th17 cells among CD4+ T lymphocytes following acupuncture contributes to its anti-inflammatory and mental health benefits [54].

Acupuncture also potentially affects neural plasticity in critical brain areas such as the hippocampus and medial prefrontal cortex (mPFC), essential for emotional regulation and depression management. Studies indicate that acupuncture may enhance resting-state functional connectivity in these areas, offering a possible mechanism for its benefits against depression [55]. Electroacupuncture, in particular, is speculated to modulate 5-HT receptors, influencing synaptic plasticity in the hippocampus and potentially improving mood [56]. These mood enhancements might also involve an increase in BDNF expression, promoting synaptic connectivity and neural regeneration, amplifying acupuncture’s antidepressant effects [57].

This study acknowledges certain limitations. Firstly, despite a thorough literature search, a paucity of studies compared acupuncture with psychotropic medications, potentially widening confidence intervals and undermining the precision of the outcomes. Secondly, as all the included studies originated from China, the findings might exhibit certain regional traits. Lastly, the quality of the studies included is rather varied, characterized by significant heterogeneity. Despite conducting subgroup analyses using different control methods, only a few groups displayed reduced heterogeneity to a statistically insignificant level. Thus, we hypothesize that the primary source of this heterogeneity might be the acupuncture treatment itself, especially the inconsistency in the selection of acupoints and the variation in treatment durations. This suggests that future research should focus on standardizing acupuncture treatment protocols, in order to reduce heterogeneity and enhance the quality of the studies. Such standardization would enable researchers to more accurately assess the efficacy and safety of acupuncture treatment.

Evidence from current clinical studies indicates that acupuncture could be a viable therapeutic option for the treatment of depression and anxiety associated with functional dyspepsia. Its therapeutic efficacy seems comparable to that of psychotropic medications. However, the quality of the included studies is not ideal, and there is considerable heterogeneity. Consequently, our conclusions need further validation from additional large-scale, multicenter, high-quality RCTs.

## Supporting information

S1 Checklist PRISMA checklist. (DOCX)

S1 File (PDF)
