# Supplementary material for: Efficacy of acupuncture for anxiety and depression in functional dyspepsia: A systematic review and meta-analysis
Source: PLoS One. 2024 Mar 7;19(3):e0298438. doi: 10.1371/journal.pone.0298438 (PMC10919749; doi:10.1371/journal.pone.0298438)
Supplement: S1 File — (PDF) [file pone.0298438.s002.pdf]

Supplemental Table 1. PubMed search strategy

Database	#1	"Dyspepsia"[Mesh]
Pubmed	#2	((((((Dyspepsias[Title/Abstract]) OR (Indigestion[Title/Abstract])) OR (Indigestions[Title/Abstract])) OR (Functional dyspepsia[Title/Abstract])) OR (non-ulcer dyspepsia[Title/Abstract])) OR (postprandial distress syndrome[Title/Abstract])) OR (epigastric pain syndrome[Title/Abstract])
	#3	#1 OR #2
	#4	"Acupuncture"[Mesh]
	#5	(((((((((((((Pharmacoacupuncture[Title/Abstract]) OR (Acupotomy Title/Abstract])) OR (Acupotomies[Title/Abstract])) OR (Pharmacopuncture Treatment[Title/Abstract])) OR (Needle[Title/Abstract])) OR (Needling[Title/Abstract])) OR (electroacupuncture[Title/Abstract])) OR (electro-acupuncture[Title/Abstract])) OR (auricular acupuncture[Title/Abstract])) OR (Ear Acupuncture[Title/Abstract])) OR (warm needle[Title/Abstract])) OR (Moxibustion[Title/Abstract])) OR (Acupuncture Points[Title/Abstract])) OR (Acupuncture Therapy[Title/Abstract])
	#6	#4 OR #5
	#7	randomized controlled trial[Publication Type] OR randomized[Title/Abstract] OR placebo[Title/Abstract]
	#8	#3 AND #6 AND #7
Embase	#1	'Dyspepsias':ab,ti OR 'Indigestion':ab,ti OR 'Indigestions':ab,ti OR 'Functional dyspepsia':ab,ti OR 'non-ulcer dyspepsia':ab,ti OR 'postprandial distress syndrome':ab,ti OR 'epigastric pain syndrome':ab,ti
	#2	'Pharmacoacupuncture':ab,ti OR 'Acupotomy':ab,ti OR 'Acupotomies':ab,ti OR 'Pharmacopuncture Treatment':ab,ti OR 'Needle':ab,ti OR 'Needling':ab,ti OR 'electroacupuncture':ab,ti OR 'electro-acupuncture':ab,ti OR 'auricular acupuncture':ab,ti OR 'Acupunctures, Ear':ab,ti OR 'Ear Acupuncture':ab,ti OR 'warm needle':ab,ti OR 'Moxibustion':ab,ti OR 'Acupuncture Points':ab,ti OR 'Acupuncture Therapy':ab,ti
	#3	'randomized controlled trial':ab,ti OR 'randomized':ab,ti OR 'placebo':ab,ti OR 'RCT':ab,ti
	#4	#1 AND #2 AND #3
Web of science	#1	TS=(Dyspepsia OR Dyspepsias OR Indigestion OR Indigestions OR Functional dyspepsia OR non-ulcer dyspepsia OR postprandial distress syndrome OR epigastric pain syndrome)
	#2	TS=(Acupuncture OR Pharmacoacupuncture OR Acupotomy OR Acupotomies OR Pharmacopuncture Treatment OR Needle OR Needling OR electroacupuncture OR electro-acupuncture OR auricular acupuncture OR Acupunctures, Ear OR Ear Acupuncture OR warm needle OR Moxibustion OR Acupuncture Points OR Acupuncture Therapy)
	#3	TS=(randomized controlled trial OR randomized OR placebo OR RCT)
	#4	#1 AND #2 AND #3
The Cochrane library	#1	(Dyspepsias):ab,ti,kw OR (Indigestion):ab,ti,kw OR (Indigestions):ab,ti,kw OR (Functional dyspepsia):ab,ti,kw OR (non-ulcer dyspepsia):ab,ti,kw OR (postprandial distress syndrome):ab,ti,kw OR (epigastric pain syndrome):ab,ti,kw
	#2	(Pharmacoacupuncture):ab,ti,kw OR(Acupotomy):ab,ti,kw OR (Acupotomies):ab,ti,kw OR(Pharmacopuncture Treatment):ab,ti,kw OR (Needle):ab,ti,kw

		OR(Needling):ab,ti,kw OR (electroacupuncture):ab,ti,kw OR(electro-acupuncture):ab,ti,kw OR (auricular acupuncture):ab,ti,kw OR(Acupunctures, Ear):ab,ti,kw OR (Ear Acupuncture):ab,ti,kw OR(warm needle):ab,ti,kw OR (Moxibustion):ab,ti,kw OR(Acupuncture Points):ab,ti,kw
	#3	(randomized controlled trial):ab,ti,kw OR(randomized):ab,ti,kw OR (placebo):ab,ti,kw OR(RCT):ab,ti,kw
	#4	# 1 AND #2 AND #3
**CNKI**	#1	篇关摘 = 消化不良 + 非溃疡性消化不良 + 非溃疡消化不良 + 功能性消化不良 + 餐后窘迫综合征 + 上腹疼痛综合征 + FD
	#2	篇关摘 =针灸 + 针刺 + 手针 + 体针 + 耳针 + 电针 + 火针 + 温针 + 艾灸 + 灸法 + 针 + 灸 + 穴 + 埋线
	#3	篇关摘 =随机对照实验 + 随机对照试验 + RCT + 随机对照 + 随机
	#4	#1 AND #2 AND #3
**WanFang**	#1	主题 = 消化不良 + 非溃疡性消化不良 + 非溃疡消化不良 + 功能性消化不良 + 餐后窘迫综合征 + 上腹疼痛综合征 + FD
	#2	主题 =针灸 + 针刺 + 手针 + 体针 + 耳针 + 电针 + 火针 + 温针 + 艾灸 + 灸法 + 针 + 灸 + 穴 + 埋线
	#3	主题 =随机对照实验 + 随机对照试验 + RCT + 随机对照 + 随机
	#4	#1 AND #2 AND #3
**VIP**	#1	题目或关键词 = 消化不良 OR 非溃疡性消化不良 OR 非溃疡消化不良 OR 功能性消化不良 OR 餐后窘迫综合征 OR 上腹疼痛综合征 OR FD
	#2	题目或关键词=针灸 OR 针刺 OR 手针 OR 体针 OR 耳针 OR 电针 OR 火针 OR 温针 OR 艾灸 OR 灸法 OR 针 OR 灸 OR 穴 OR 埋线
	#3	题目或关键词=随机对照实验 OR 随机对照试验 OR RCT OR 随机对照 OR 随机
	#4	#1 AND #2 AND #3
**CBM**	#1	"消化不良"[不加权:扩展]
	#2	"功能性消化不良"[常用字段:智能] OR "非溃疡性消化不良"[常用字段:智能] OR "非溃疡消化不良"[常用字段:智能] OR "上腹疼痛综合征"[常用字段:智能] OR "餐后窘迫综合征"[常用字段:智能] OR "FD"[常用字段:智能]
	#3	(#2) OR (#1)
	#4	((((((((("针灸疗法"[不加权:扩展]) OR "温针疗法"[不加权:扩展]) OR "电针"[不加权:扩展]) OR "火针疗法"[不加权:扩展]) OR "灸法"[不加权:扩展]) OR "埋线"[不加权:扩展]) OR "穴位贴敷法"[不加权:扩展]) OR "耳穴贴压"[不加权:扩展]) OR "推拿"[不加权:扩展]) OR "针刺疗法"[不加权:扩展]
	#5	"针刺"[常用字段:智能] OR "电针"[常用字段:智能] OR "火针"[常用字段:智能] OR "温针"[常用字段:智能] OR "艾灸"[常用字段:智能] OR "埋线"[常用字段:智能] OR "贴敷"[常用字段:智能] OR "耳穴"[常用字段:智能] OR "推拿"[常用字段:智能] OR "中医外治"[常用字段:智能]
	#6	(#5) OR (#4)
	#7	随机对照试验[不加权:扩展]
	#8	"随机对照试验"[常用字段:智能] OR "随机对照实验"[常用字段:智能] OR "随机对照研究"[常用字段:智能] OR "随机对照"[常用字段:智能] OR "随机"[常用字段:智能] OR "RCT"[常用字段:智能]
	#9	(#8) OR (#7)
	#10	(#9) AND (#6) AND (#3)

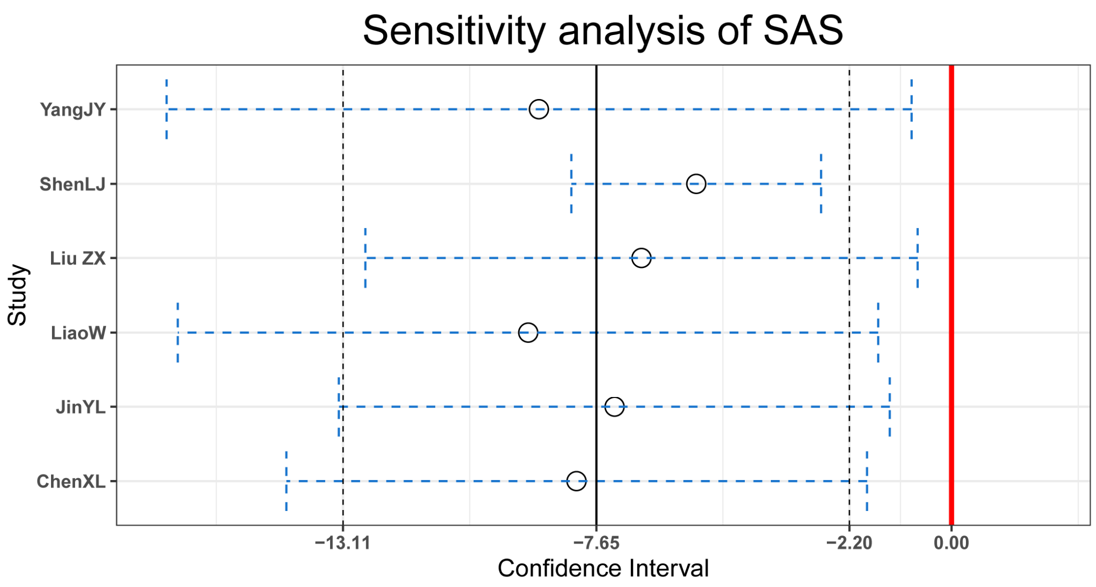

Supplemental Fig 1. Sensitivity analysis of SAS

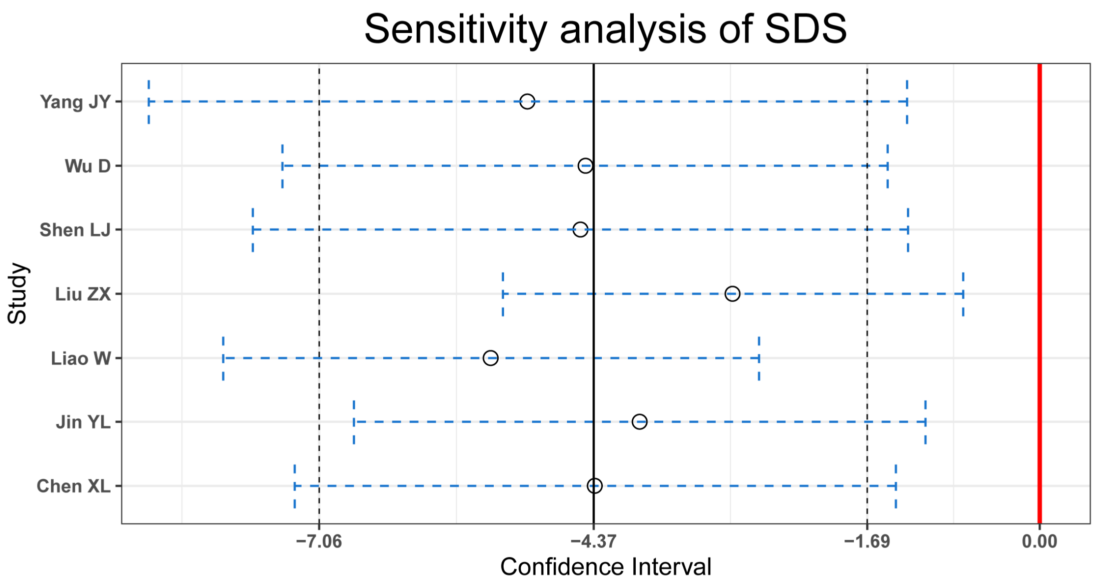

Supplemental Fig 2. Sensitivity analysis of SDS

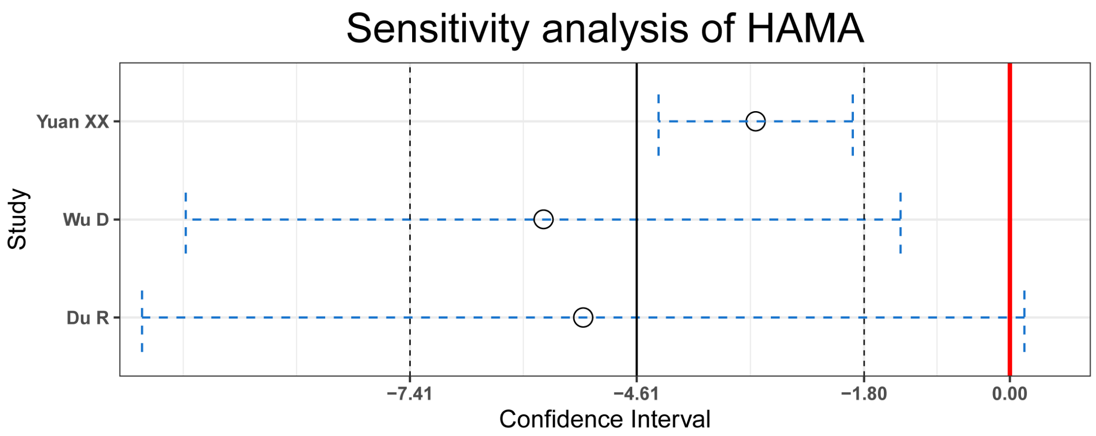

Supplemental Fig 3. Sensitivity analysis of HAMA

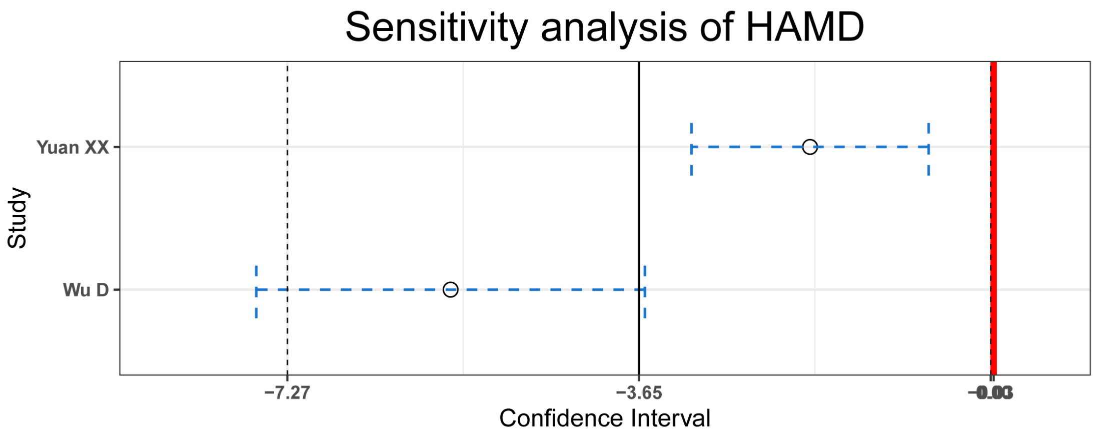

Supplemental Fig 4. Sensitivity analysis of HAMD

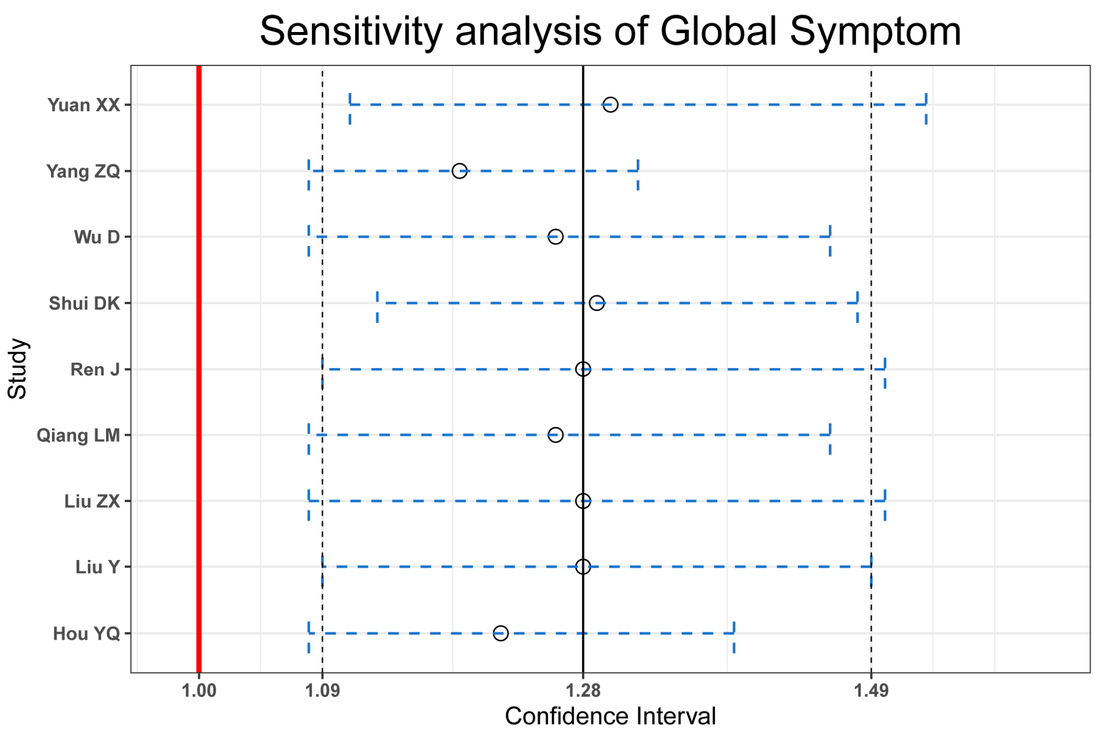

Supplemental Fig 5. Sensitivity analysis of Global Symptom

SAS for anxiety and depression in functional dyspepsia

Patient or population: patients with anxiety and depression in functional dyspepsia
Settings:
Intervention: SAS

Outcomes	Illustrative comparative risks* (95% CI)		Relative effect (95% CI)	No of Participants (studies)	Quality of the evidence (GRADE)	Comments
	Assumed risk	Corresponding risk				
	Control	SAS				
**SAS_sub - Acupuncture vs. First-Line**		The mean sas_sub - acupuncture vs. first-line in the intervention groups was **8.89 lower** (22.86 lower to 5.09 higher)		130 (2 studies)	⊕⊖⊖⊖ **very low**[1,2,3,4]	
**SAS_sub - Acupuncture vs. First-line+Second-line**		The mean sas_sub - acupuncture vs. first-line+second-line in the intervention groups was **6.49 lower** (18.18 lower to 5.2 higher)		180 (2 studies)	⊕⊖⊖⊖ **very low**[1,2,3,4]	
**SAS_sub - Acupuncture vs. Placebo Acupuncture**		The mean sas_sub - acupuncture vs. placebo acupuncture in the intervention groups was **7.07 lower** (11.03 to 3.1 lower)		83 (2 studies)	⊕⊖⊖⊖ **very low**[2,3,5,6]	

*The basis for the **assumed risk** (e.g. the median control group risk across studies) is provided in footnotes. The **corresponding risk** (and its 95% confidence interval) is based on the assumed risk in the comparison group and the **relative effect** of the intervention (and its 95% CI).

CI: Confidence interval;

GRADE Working Group grades of evidence
High quality: Further research is very unlikely to change our confidence in the estimate of effect.
Moderate quality: Further research is likely to have an important impact on our confidence in the estimate of effect and may change the estimate.
Low quality: Further research is very likely to have an important impact on our confidence in the estimate of effect and is likely to change the estimate.
Very low quality: We are very uncertain about the estimate.

[1] Blind method not described in detail
[2] No detailed description of random methods or allocation of hidden methods
[3] heterogeneity
[4] Confidence interval is too wide
[5] Per-Protocol analysis
[6] Publication Bias

Supplemental Fig 6. GRADE assessment for the SAS

SDS for anxiety and depression in functional dyspepsia

Patient or population: patients with anxiety and depression in functional dyspepsia
Settings:
Intervention: SDS

Outcomes	Illustrative comparative risks* (95% CI)		Relative effect (95% CI)	No of Participants (studies)	Quality of the evidence (GRADE)	Comments
	Assumed risk	Corresponding risk				
	Control	SDS				
**SDS_sub - Acupuncture vs. First-Line**		The mean sds_sub - acupuncture vs. first-line in the intervention groups was **2.71 lower** (5.19 to 0.23 lower)		130 (2 studies)	⊕⊖⊖⊖ **very low**[1,2,3]	
**SDS_sub - Acupuncture vs. First-line+Second-line**		The mean sds_sub - acupuncture vs. first-line+second-line in the intervention groups was **5.02 lower** (17.4 lower to 7.36 higher)		180 (2 studies)	⊕⊖⊖⊖ **very low**[1,2,3,4]	
**SDS_sub - Acupuncture vs. Placebo Acupuncture**		The mean sds_sub - acupuncture vs. placebo acupuncture in the intervention groups was **4.63 lower** (6.28 to 2.98 lower)		173 (3 studies)	⊕⊕⊖⊖ **low**[1,2,5]	

*The basis for the **assumed risk** (e.g. the median control group risk across studies) is provided in footnotes. The **corresponding risk** (and its 95% confidence interval) is based on the assumed risk in the comparison group and the **relative effect** of the intervention (and its 95% CI).

CI: Confidence interval;

GRADE Working Group grades of evidence
High quality: Further research is very unlikely to change our confidence in the estimate of effect.
Moderate quality: Further research is likely to have an important impact on our confidence in the estimate of effect and may change the estimate.
Low quality: Further research is very likely to have an important impact on our confidence in the estimate of effect and is likely to change the estimate.
Very low quality: We are very uncertain about the estimate.

[1] Blind method not described in detail
[2] No detailed description of random methods or allocation of hidden methods
[3] heterogeneity
[4] Confidence interval is too wide
[5] Per-Protocol analysis

Supplemental Fig 7. GRADE assessment for the SDS

HAMA for anxiety and depression in functional dyspepsia

Patient or population: patients with anxiety and depression in functional dyspepsia
Settings:
Intervention: HAMA

Outcomes	Illustrative comparative risks* (95% CI)		Relative effect (95% CI)	No of Participants (studies)	Quality of the evidence (GRADE)	Comments
	Assumed risk	Corresponding risk				
	Control	HAMA				
**HAMA_sub - Acupuncture vs. First-Line**		The mean hama_sub - acupuncture vs. first-line in the intervention groups was **5.76 lower** (10.18 to 1.35 lower)		158 (2 studies)	⊕⊖⊖⊖ **very low**[1,2,3]	
**HAMA_sub - Acupuncture vs. Placebo Acupuncture**		The mean hama_sub - acupuncture vs. placebo acupuncture in the intervention groups was **2.58 lower** (4.33 to 0.83 lower)		90 (1 study)	⊕⊖⊖⊖ **very low**[1,2,4]	

*The basis for the **assumed risk** (e.g. the median control group risk across studies) is provided in footnotes. The **corresponding risk** (and its 95% confidence interval) is based on the assumed risk in the comparison group and the **relative effect** of the intervention (and its 95% CI).

CI: Confidence interval;

GRADE Working Group grades of evidence
High quality: Further research is very unlikely to change our confidence in the estimate of effect.
Moderate quality: Further research is likely to have an important impact on our confidence in the estimate of effect and may change the estimate.
Low quality: Further research is very likely to have an important impact on our confidence in the estimate of effect and is likely to change the estimate.
Very low quality: We are very uncertain about the estimate.

[1] Blind method not described in detail
[2] No detailed description of random methods or allocation of hidden methods
[3] heterogeneity
[4] Imprecision due to less research

Supplemental Fig 8. GRADE assessment for the HAMA

HAMD for anxiety and depression in functional dyspepsia

Patient or population: patients with anxiety and depression in functional dyspepsia
Settings:
Intervention: HAMD

Outcomes	Illustrative comparative risks* (95% CI)		Relative effect (95% CI)	No of Participants (studies)	Quality of the evidence (GRADE)	Comments
	Assumed risk	Corresponding risk				
	Control	HAMD				
**HAMD_sub - Acupuncture vs. First-Line**		The mean hamd_sub - acupuncture vs. first-line in the intervention groups was **5.59 lower** (7.59 to 3.59 lower)		63 (1 study)	⊕⊝⊝⊝ **very low**[1,2,3]	
**HAMD_sub - Acupuncture vs. Placebo Acupuncture**		The mean hamd_sub - acupuncture vs. placebo acupuncture in the intervention groups was **1.89 lower** (3.11 to 0.67 lower)		90 (1 study)	⊕⊕⊝⊝ **low**[1,3]	

*The basis for the **assumed risk** (e.g. the median control group risk across studies) is provided in footnotes. The **corresponding risk** (and its 95% confidence interval) is based on the assumed risk in the comparison group and the **relative effect** of the intervention (and its 95% CI).

CI: Confidence interval;

GRADE Working Group grades of evidence
High quality: Further research is very unlikely to change our confidence in the estimate of effect.
Moderate quality: Further research is likely to have an important impact on our confidence in the estimate of effect and may change the estimate.
Low quality: Further research is very likely to have an important impact on our confidence in the estimate of effect and is likely to change the estimate.
Very low quality: We are very uncertain about the estimate.

[1] Blind method not described in detail
[2] No detailed description of random methods or allocation of hidden methods
[3] Imprecision due to less research

Supplemental Fig 9. GRADE assessment for the HAMD

HADS for anxiety and depression in functional dyspepsia

Patient or population: patients with anxiety and depression in functional dyspepsia
Settings:
Intervention: HADS

Outcomes	Illustrative comparative risks* (95% CI)		Relative effect (95% CI)	No of Participants (studies)	Quality of the evidence (GRADE)	Comments
	Assumed risk	Corresponding risk				
	Control	HADS				
**HADS**		The mean hads in the intervention groups was **1 lower** (2.65 lower to 0.65 higher)		278 (1 study)	⊕⊕⊕⊝ **moderate**[1]	

*The basis for the **assumed risk** (e.g. the median control group risk across studies) is provided in footnotes. The **corresponding risk** (and its 95% confidence interval) is based on the assumed risk in the comparison group and the **relative effect** of the intervention (and its 95% CI).

CI: Confidence interval;

GRADE Working Group grades of evidence
High quality: Further research is very unlikely to change our confidence in the estimate of effect.
Moderate quality: Further research is likely to have an important impact on our confidence in the estimate of effect and may change the estimate.
Low quality: Further research is very likely to have an important impact on our confidence in the estimate of effect and is likely to change the estimate.
Very low quality: We are very uncertain about the estimate.

[1] Imprecision due to less research

Supplemental Fig 10. GRADE assessment for the HADS

Efficiency for anxiety and depression in functional dyspepsia

Patient or population: patients with anxiety and depression in functional dyspepsia
Settings:
Intervention: Efficiency

Outcomes	Illustrative comparative risks* (95% CI)		Relative effect (95% CI)	No of Participants (studies)	Quality of the evidence (GRADE)	Comments
	Assumed risk	Corresponding risk				
	Control	Efficiency				
**Efficiency_sub - Acupuncture vs. First-Line**	**Study population**		**RR 1.11** (1.02 to 1.21)	335 (5 studies)	⊕⊕⊖⊖ **low**[1,2]	
	**804 per 1000**	**892 per 1000** (820 to 972)				
	**Moderate**					
	**820 per 1000**	**910 per 1000** (836 to 992)				
**Efficiency_sub - Acupuncture vs. First-line+Second-line**	**Study population**		**RR 1.24** (1.04 to 1.47)	100 (1 study)	⊕⊖⊖⊖ **very low**[1,2,3]	
	**760 per 1000**	**942 per 1000** (790 to 1000)				
	**Moderate**					
	**760 per 1000**	**942 per 1000** (790 to 1000)				
**Efficiency_sub - Acupuncture vs. Placebo Acupuncture**	**Study population**		**RR 1.72** (1.14 to 2.61)	208 (3 studies)	⊕⊖⊖⊖ **very low**[1,2,4,5]	
	**524 per 1000**	**902 per 1000** (598 to 1000)				
	**Moderate**					
	**433 per 1000**	**745 per 1000** (494 to 1000)				

*The basis for the **assumed risk** (e.g. the median control group risk across studies) is provided in footnotes. The **corresponding risk** (and its 95% confidence interval) is based on the assumed risk in the comparison group and the **relative effect** of the intervention (and its 95% CI).

CI: Confidence interval; **RR:** Risk ratio;

GRADE Working Group grades of evidence
High quality: Further research is very unlikely to change our confidence in the estimate of effect.
Moderate quality: Further research is likely to have an important impact on our confidence in the estimate of effect and may change the estimate.
Low quality: Further research is very likely to have an important impact on our confidence in the estimate of effect and is likely to change the estimate.
Very low quality: We are very uncertain about the estimate.

1 Blind method not described in detail
2 No detailed description of random methods or allocation of hidden methods
3 Imprecision due to less research
4 Per-Protocol analysis
5 heterogeneity

Supplemental Fig 11. GRADE assessment for the Global Symptom
